# Double feature selection and cluster analyses in mining of microarray data from cotton

**DOI:** 10.1186/1471-2164-9-295

**Published:** 2008-06-20

**Authors:** Magdy S Alabady, Eunseog Youn, Thea A Wilkins

**Affiliations:** 1 Functional Genomics Lab, Department of Plant and Soil Science, Texas Tech University, Lubbock, Texas 79409, USA; 2 Department of Computer Science, Texas Tech University, Lubbock, Texas 79409, USA

## Abstract

**Background:**

Cotton fiber is a single-celled seed trichome of major biological and economic importance. In recent years, genomic approaches such as microarray-based expression profiling were used to study fiber growth and development to understand the developmental mechanisms of fiber at the molecular level. The vast volume of microarray expression data generated requires a sophisticated means of data mining in order to extract novel information that addresses fundamental questions of biological interest. One of the ways to approach microarray data mining is to increase the number of dimensions/levels to the analysis, such as comparing independent studies from different genotypes. However, adding dimensions also creates a challenge in finding novel ways for analyzing multi-dimensional microarray data.

**Results:**

Mining of independent microarray studies from Pima and Upland (TM1) cotton using double feature selection and cluster analyses identified species-specific and stage-specific gene transcripts that argue in favor of discrete genetic mechanisms that govern developmental programming of cotton fiber morphogenesis in these two cultivated species. Double feature selection analysis identified the highest number of differentially expressed genes that distinguish the fiber transcriptomes of developing Pima and TM1 fibers. These results were based on the finding that differences in fibers harvested between 17 and 24 day post-anthesis (dpa) represent the greatest expressional distance between the two species. This powerful selection method identified a subset of genes expressed during primary (PCW) and secondary (SCW) cell wall biogenesis in Pima fibers that exhibits an expression pattern that is generally reversed in TM1 at the same developmental stage. Cluster and functional analyses revealed that this subset of genes are primarily regulated during the transition stage that overlaps the termination of PCW and onset of SCW biogenesis, suggesting that these particular genes play a major role in the genetic mechanism that underlies the phenotypic differences in fiber traits between Pima and TM1.

**Conclusion:**

The novel application of double feature selection analysis led to the discovery of species- and stage-specific genetic expression patterns, which are biologically relevant to the genetic programs that underlie the differences in the fiber phenotypes in Pima and TM1. These results promise to have profound impacts on the ongoing efforts to improve cotton fiber traits.

## Background

Microarray technology provides data in high-dimensional space defined by the size of the genome under investigation. With such high-dimensional data, feature selection methods are essentially classification tools used to identify gene clusters that reveal biologically meaningful relationships [1]. A classical use of feature selection analysis [2] is to identify the most discriminating features or dimension in a matrix of microarray data [3]. Developing new methods to discriminate between sets of microarray data for both dimensions (time points/conditions) and features (genes) will improve data mining processes that in turn will lead to the discovery of biologically relevant relationships. In cotton fiber genomics, microarrays provide a robust technology for identifying developmentally regulated genes during cotton fiber morphogenesis in the two major cultivated species, *G. barbadense *L. cv. Pima S7 (Gb) and *G. hirsutum *L. cv. TM1 (Gh). These two species vary in fiber characteristics and yield; *G. barbadense *offers superior fiber quality properties like length, fineness, and strength, while *G. hirsutum *is characterized by high yield. Breeding programs around the world are working towards developing high-yielding *G. hirsutum *cultivars with the fiber properties of *G. barbadense*. In both species, fiber development occurs in four overlapping stages; initiation (-3 to 5 dpa), elongation (3 to 21 dpa), secondary cell wall synthesis (14 to 45 dpa), and maturation (40 to 55 dpa) [4]. Despite the similarity in timing and duration of developmental stages, however, inherent differences in the developmental programs lead to the production of fiber with discrete phenotypic differences. Therefore, elucidating the genetic mechanisms that underlie these differences is crucial to designing strategies for the genetic enhancement of cotton fiber traits with superior Pima characteristics. In this respect, transcriptome profiling of developing Gb and Gh fibers is pivotal to discovering the specific genetic program that drives the development of fiber in these genotypes. Of more importance is the identification of the developmental signals that trigger differential regulation of biological processes that yield the discrete Gb and Gh phenotypes. Few studies to date were conducted to study fiber genomics at the developmental level in a single cotton species (Reviewed in [5,6]), and no studies have focused on molecular differences between both species (Gb and Gh) at the transcriptional level. In our lab, stage-specific developmentally regulated genes during fiber morphogenesis were identified independently in Pima and TM1 species (Alabady and Wilkins, In Preparation). In this study, we describe a novel application of feature selection analysis to simultaneously select between both features (genes) and dimensions (time points) of the developmental transcriptome of the two species. This novel application is termed "double feature analysis" as it enables simultaneous selection between features and dimensions in an unsupervised learning context, and therefore differs from more traditional feature selection, which selects within only one variable.

The objectives of this study were to 1) Discover the top discriminating genes between the transcriptomes of Pima and TM1 fibers at the most distant developmental time points using the novel application of double feature selection (DFS) analysis, and 2) Determine the intersecting genes between developmentally regulated fiber profiles (Pima and TM1) and the top differentially expressed genes identified by DFS, and to link the intersected genes to specific stages of fiber development.

## Results

### Reliability of the Microarray data and design

**Figure 1:**
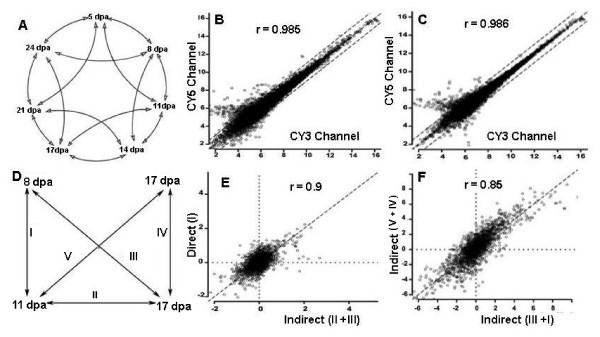
**Assessment of the quality of double loop design-derived microarray data**. **A**. Diagram of microarray double loop design comparing gene expression at 7 developmental time points. **B **and **C**. Scatter plots showing the high correlation between both dye channels in self-hybridization control experiments at 11 and 14 dpa time points. **D**. Routes of direct and indirect comparisons between 11 and 14 dpa. **E **and **F**. Scatter plots showing the correlation between expression data between direct vs. indirect routes, and indirect vs. indirect routes, respectively.

A double loop microarray design (Figure 1A) with dye swap experiments was adopted to profile the transcriptome of developing Pima and TM1 fibers independently. This design generated 8 data points (4 per each dye channel) per gene at each time point, excluding data points from duplicated spots within each array. Dye bias, an inherent problem to microarrays, identifies differential expression that is falsely attributed to the nature of the dye rather than to true differences in biological samples. In this study, data from both channels were highly correlated as the Pearson correlation coefficients between Log_2 _Cy3 signal intensity versus Log_2 _Cy5 signal intensity of the 11 and 14 dpa self-hybridization control experiments [7] were 0.985 and 0.986, respectively (Figures 1B and 1C). The linearity between data estimated from direct and indirect hybridizations (e.g., through a common reference) for the same comparison was also very high (Figures 1D and 1E). Similarly, there was a strong correlation between data obtained from two indirect routes for the same comparison (Figures 1D and 1F). Therefore, expression data in the transcriptome profiles of Pima and TM1 developing fibers were reliable and reproducible for interpreting results in a biologically relevant context.

### Developmentally regulated expression profiles in Pima and TM1

In the transcriptome of developing Pima and TM1 fibers, 2943 and 2281 genes, respectively, were significantly and differentially expressed across developmental stages. Normalized to the total number of the genes profiled in this study (12063), 17.24% and 11.75% of the genes were found to be specifically regulated in Pima and TM1, respectively, while 7.15% of the genes were regulated in both species. Among Pima developmentally-regulated genes, K-means clustering produced 10 clusters or profiles of co-expressed genes. The expression patterns of these clusters were linked to discrete developmental stages based on the correspondence between the highest level of expression within a cluster and a developmental stage. For instance, genes in clusters one and five were up-regulated only during the fiber elongation stage, and hence, were classified as expansion-associated genes during primary cell wall biogenesis, which represented 4.65% of the profiled genes (Table 1). Similarly, among the eight clusters of TM1 developmentally-regulated profiles, genes in clusters five and eight were assigned an expansion-associated role and comprised 2.95% of the profiled genes (Table 1). Genes down-regulated during both primary cell wall (PCW)/fiber expansion and secondary cell wall (SCW) synthesis relative to 5 dpa were considered to be initiation-specific, which represented 10.76% and 8.38% of profiled genes in the Pima and TM1 transcriptomes, respectively. In general, the percentage of significantly regulated genes associated with each of the developmental stages was slightly higher for all stages in Pima than in TM1, except for the cellulose synthesis-associated genes where the reverse was true (Table 1).

**Table 1:** Developmental dissection of developmentally regulated genes in Pima and TM1 fiber transcriptomes revealed species- and stage-specific clusters.

**Data sets**	**No. of genes (%)**	**No. of clusters**	**Stage specific clusters (genes)**	**Description (%)**
Pima developmentally regulated profile	2943 (**24.39**)	10	1 (323), 5 (239)	Up^1 ^during PCW synthesis only (**4.65**)
			2 (232), 9 (209)	Up^1 ^during SCW deposition only (**3.65**)
			4 (399)	Up^1 ^in PCW and SCW (**3.31**)
			3(493), 6(588), 8 (217)	Down^2 ^in PCW and SCW (**10.76**)
			7 (77), 10 (86),	Oscillating (**1.35**)

TM1 developmentally regulated profile	2281 (**18.90**)	8	5 (234), 8 (122)	Up^1 ^during PCW synthesis only (**2.95**)
			6 (160), 7 (400)	Up^1 ^during SCW deposition only (**4.64**)
			1 (353)	Up^1 ^in PCW and SCW (**2.92**)
			2(392), 3(517), 4 (103)	Down^2 ^in PCW and SCW (**8.38**)

Top differentially expressed genes between Pima and TM1 profiles of fiber transcriptome	1167 (**9.67**)	2	1 (465)	Pima specific pattern (**3.85**)
			2 (702)	TM1 specific pattern (**5.81**)

^1 ^Up-regulated, ^2 ^Down-regulated; All percentages in this table were calculated relative to the total number of profiled genes (12063 after filtration) in this study

### Differential expression profiles in Pima versus TM1 fibers

**Figure 2:**
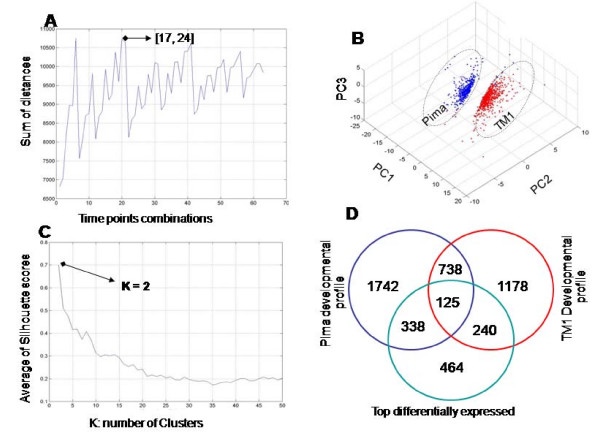
**Double feature selection analysis of Pima and TM1 transcriptome profiles of developing fibers**. **A **and **B **show that K = 2 in K-means clustering was the best value according to the Silhouette score (**A**) and degree of separation in SVD analysis (**B**). The greatest genetic distances between the two profiles were defined by 17 and 24 dpa stages (**C**). **D **shows the intersection between the top differentially expressed genes between Pima and TM1 transcriptomes and developmentally regulated profiles in Pima and TM1.

The double feature selection (DFS) analysis developed in this study identified discrete time points that distinguished developmental stages both within and between species. The maximum number of differentially expressed genes occurred coincident with peak cellulose synthesis and SCW biogenesis at 24 dpa in both species. Similarly, the greatest genetic distance between any two time points of the two species occurred during 17 and 24 dpa (Figure 2A) corresponding to the PCW/SCW transition stage. It was for these reasons these two data points (17 and 24 dpa) were used to further characterize the differences between the fiber transcriptomes of Pima and TM1. However, three other combinations of time points were found to be highly discriminating between Pima and TM1 profiles (Figure 2A), which might also participate in coding the differences between both species. The top discriminating genes (1167 genes) at 17 and 24 dpa were identified based on the expression distance between the two species with a cut-off distance of D ≥ 2. Upon clustering, two distinct clusters were created (Figure 2B) with an average Silhoutte score [8] equal to 0.7 (Figure 2C), which implies a strong assignment of genes to clusters. These two clusters were visualized using singular value decomposition (SVD) [9] as shown in Figure 2B. SVD analysis showed that the first three principal components explained 74% of the total variance in the difference of expression levels. Cluster one included 465 genes that were up-regulated in Pima developmental profiles, while the same genes were down-regulated in TM1 profiles. Cluster 2 comprised 702 genes that were specifically up-regulated in the TM1 developmental profile and down-regulated in Pima profile. Figure 2B illustrates how distinct the two clusters were from one another, indicating that these two clusters are naturally occurring within the top discriminating gene set. These results reflect the efficiency of double feature selection in mining two independent microarray studies, and extracting naturally-occurring data variances that are biologically relevant.

### Top discriminating genes partially intersect with developmentally regulated genes

Identifying the intersecting genes between developmentally regulated profiles identified by independent analysis of Pima and TM1 transcripomes and the top discriminating genes identified by DFS serves two purposes: it assesses the level of significance in the top discriminating genes, and identifies species-specific differentially regulated profiles. A custom Perl script was developed to identify intersecting genes between the following three data sets: developmentally regulated fiber genes from Pima, developmentally regulated fiber genes from TM1, and the DFS top discriminating genes (Figure 2D). Three intersecting gene sets of interest were generated. Set 1 includes 125 genes that intersect the top differentially expressed genes identified by DFS and developmental profiles (Additional file 1), and represents highly-distant differentially expressed genes between developmentally-regulated fiber profiles of Pima and TM1. Specific to the Pima developmental profile, Set 2 includes 338 genes that intersect with the top DFS differentially expressed genes, and were not significantly expressed in TM1. Similarly, Set 3 included 240 genes that intersect with TM1 developmentally regulated genes and the top DFS differentially expressed genes, and were not significantly expressed in Pima (Figure 2D). These three gene sets are anticipated to be key to elucidating the genetic mechanisms that underpin phenotypic differences in the physical fiber traits of the two species. The expression patterns of Set 1 genes in Pima fiber were completely opposite to those of TM1, and likely reflect genetic differences in developmental programming that account for the genotypic differences responsible for the fiber phenotype. Moreover, the fact that Set 1 genes are significantly regulated in a manner that is diametrically opposed in the two species suggests that the expression level of these genes might be involved in triggering/stimulation the expression of genes in Set 2 (Pima-specific) and Set 3 (TM1-specific). In this article, functional analysis of genes in Set 1 (Additional file 1) was the main focus of the discussion.

### Species and stage specific gene profiles

**Figure 3:**
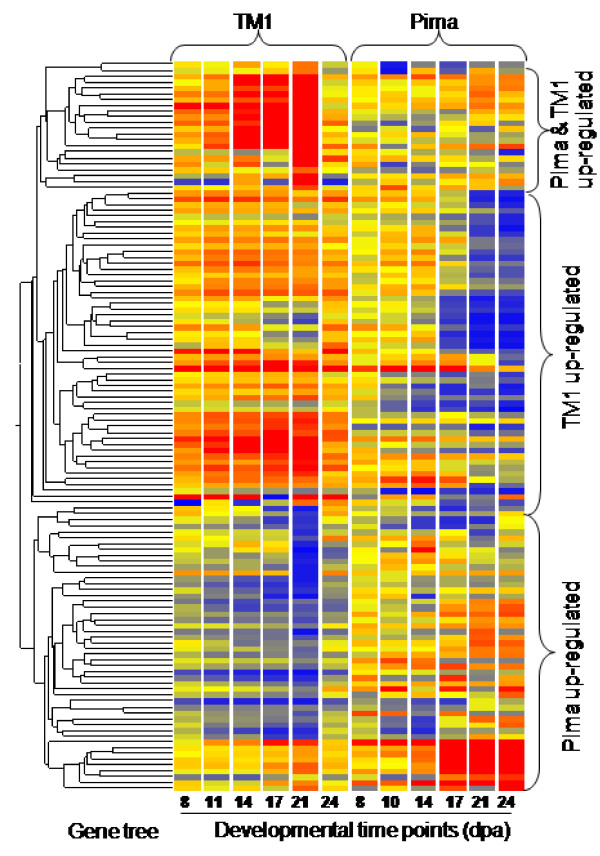
**Expression and condition tree (2 genotypes) of genes in Set 1 showing three profiles of differential regulation between Pima and TM1**. Up-regulated Pima genes were down-regulated in TM1, and vice versa for the TM1 up-regulated profile. A small group of genes was up-regulated in both genotypes. In the tree, red color indicates up-regulation, while blue color indicated down-regulation. The darker the color, the higher/lower (depending on color) level of expression of the corresponding gene.

Genes in Set 1 are anticipated to play key roles in fiber morphogenesis and to exert a direct impact on fiber development and the phenotypic differences between Pima and TM1 fibers, and therefore warrant further investigation within a biologically relevant context. Within Set 1, three species-specific regulatory trends were identified: genes up-regulated in Pima but down-regulated in TM1, up-regulated in TM1 and down-regulated in Pima, and up-regulated in both species (Figure 3). Further dissection of these genes using K-means clustering revealed five species-specific and developmental stage-specific clusters of co-expressed genes (Table 2). Interestingly, the expression profiles of these clusters revealed that a major difference in gene expression dynamics in the developmental programs of Pima and TM1 occurs mainly in the intervals between 14 to 21 dpa, and 21 to 24 dpa. These intervals correspond to the transition period overlapping PCW and SCW synthesis, and coincide with the termination of rapid polar elongation and early entry into cellulose biosynthesis and biogenesis of the SCW [4], respectively. Approximately 34.4% (43) of the genes in this data set are of unknown function or annotated as hypothetical proteins. Therefore, gene ontology and pathway analyses were limited to 65.6% (82) of the genes in Set 1. Stage-specific and species-specific metabolic/regulatory pathways and molecular functions identified for genes in each cluster specifically characterize each fiber developmental stage at the molecular level. For instance, genes in cluster 3 were up-regulated during the synthesis of both PCW and SCW in developing Pima fibers, while they were down-regulated during the same stages in TM1 (Table 2). In this cluster, sucrose metabolism was the top represented GO category (*P *= 0.03) and pathway (Table 2). This finding highlights the important role of sucrose metabolism in fiber morphogenesis and as a diagnostic marker for the structural properties that distinguish the superior Pima fiber phenotype. Interestingly, the top represented GO category in cluster 5 was phenylalanine (PAL) metabolism (*P *= 0.004), which included genes up-regulated during PCW synthesis in TM1 and SCW deposition in Pima, but down-regulated during the transition stage PCW/SCW in both species (Table 2). In contrast to PAL metabolism, the coumarin biosynthetic pathway, the major pathway in cluster 2 (*P *= 0), was down-regulated during Pima SCW biogenesis and up-regulated during the PCW/SCW transition stage in TM1. The up-regulation of phenolic synthesis pathways during PCW development and the PCW/SCW transition stages in TM1 suggests a possible role in curtailing the rate of PCW expansion in TM1 relative to developing Pima fibers.

**Table 2:** Developmental, function and pathway analyses of 125 cotton fiber genes (Set 1) identified by expression profiling and double feature selection analysis highlighting the top represented GO pathways.

**Cluster (genes)**	**Pima pattern**	**TM1 pattern**	**Top pathway (*P *value)**	**Top GO (*P *value)**
1 (13)	Slightly up in SCW	Highly up in SCW	Reductive carboxylase cycle **(0.013)**	Response to biotic stimulus **(0.02)**
2 (34)	Down in SCW	Slightly up in the overlapped transition from PCW to SCW	Stilbene, cumarine and lignin biosynthesis **(0)**	Purine nucleotide binding **(0.025)**
3 (43)	Up in both PCW and SCW	Down in both PCW and SCW	Starch and Sucrose metabolism **(0.03)**	Sucrose metabolism **(0.02)**
4 (28)	Up in PCW and down in SCW	Up in both PCW and SCW	VEGF signaling pathway **(0.02)**	Serine-type peptidase activity **(0.0003)**
5 (7)	Down in the transition from PCW to SCW and up in SCW	Up in the PCW and down in the transition from PCW to SCW	Phenylalanine metabolism **(0.004)**	Transferase activity **(0.01)**

## Discussion

Although developmental programming of fiber morphogenesis is very similar in both Pima and TM1, species-specific differences in the structure and composition of the cell walls produce discrete fiber phenotypes and fiber quality traits. It is crucial to discover the genes responsible for governing fiber properties, and especially those diagnostic genes of the Pima fiber phenotype for the genetic enhancement of fiber quality. The main objective here was to apply a novel application of double feature selection analysis to microarray data as a means for identifying transcriptional differences between developing Pima and TM1 fibers, and to gain novel insight into the mechanisms that underlie the phenotypic differences. From a global perspective, developmentally regulated genes during fiber morphogenesis only partially overlap in Pima and TM1, indicating that discrete genetic mechanisms govern fiber morphogenesis are involved. The higher percentage of the transcriptome that is specifically regulated in developing Pima fiber (17.24%) relative to TM1 (11.75%) is consistent with genetic mapping studies showing a decided bias for Pima alleles in interspecific mapping populations [10]. Moreover, the portions of the transcriptome contributing to each of the developmental stages are higher in Pima than in TM1 for all stages of fiber development, with the surprising exception of the SCW stage, in which 4.64% of the transcriptome is specific to SCW synthesis in TM1 as opposed to only 3.65% in Pima (Table 1). Keeping in mind the superiority of Pima fiber quality relative to TM1 appears to be primarily controlled by cellulose synthesis and associated metabolic processes during the SCW biogenesis lends even greater credence to our findings. This study provides the first compelling evidence that developmental programming of fiber morphogenesis in Pima and Upland cottons are linked to discrete genetic mechanisms that govern the fiber phenotype (Table 1). Such information will prove vital to molecular breeding programs that focus on the genetic enhancement of fiber quality. The identification of developmentally-regulated, stage- and species-specific gene clusters in this study is the first step toward developing a comprehensive understanding of fiber development at the molecular genetics level. Moreover, genetic dissection of the fiber transcriptome into stage- and species-specific profiles paves the way for addressing important biological questions in plant cell development and applications in agricultural biotechnology to improve fiber traits.

The novel application of feature selection analysis established that the most distant time points, in terms of gene expression, between Pima and TM1 development occur at 17 and 24 dpa (Figure 2A). With a small genotype-based variation in developmental timing and duration of the PCW/SCW transition stage, the interval between 17 and 24 dpa represents the general frame for PCW/SCW transition stage. The duration of this stage is thought to be species-specific and longer in species that produces longer fibers [11], which will have direct influence in both fiber length and strength. This explains the importance of indentifying top discriminating genes at these two time points between the transcriptome of the two genotypes. Based on expression profiles of the fiber transcriptome at these two points, the top discriminating genes (1167) between Pima and TM1 are naturally classified into Pima-specific (3.85%) and TM1-specific (5.81%) as identified by K-means clustering and supported by SVD analysis (Figures 2B and 2C). The reliability and biological relevance of the results prove that double feature selection analysis offers a powerful new tool with applications in data mining of microarray data. Species-specific regulation patterns represented 60.21% of the top discriminating genes: 28.96% of which are Pima-specific, 20.56% TM1-specific, and 10.71% are regulated in both Pima and TM1 (Figure 2D). The results suggest that genes that intersect in the three data sets are major players of functional importance in fiber developmental programs that in turn, dictate the physical differences between Pima and TM1 fiber phenotypes. Interestingly, expression patterns of Set 1 genes (10.71%) in Pima fibers are the opposite of the patterns produced in TM1, leading to the speculation that expression of Set 1 genes influences, either directly or indirectly, the execution of genetic programs encoded by gene Sets 2 (28.96%) and 3 (20.56%) in Pima and TM1, respectively. Therefore, it is possible that differential expression of Set 1 genes between Pima and TM1 may trigger or stimulate the expression of genes in Sets 2 and 3 in a species-specific manner. Thus, Set 1 genes may be key to the successful genetic manipulation of cotton fiber traits. Further classification of Set 1 genes identified five species- and stage-specific gene clusters regulated primarily at the transition stage between PCW and SCW synthesis, and the early phases of SCW biogenesis (Table 2). Functional analysis of these five species- and stage-specific clusters unveiled major pathways that are differentially regulated in Pima in contrast to TM1 fibers. Cotton fiber cell walls are >96% cellulose at maturity and differential regulation of carbohydrate metabolism is no doubt a critical component in determining the fiber phenotype, which in turn, hinges on the structure and composition of the PCW and SCW. Therefore, the fact that sucrose metabolism (*p *= 0.02) is up-regulated during PCW and SCW in Pima versus TM1 and is associated with higher sucrose synthase activity is an important discovery. Sucrose synthase is thought to interact with the cellulose synthase complex to presumably act as a metabolic channel to convert sucrose into glucose via UDP-glucose to add sugar moieties to the growing glucan chain during cellulose synthesis [12]. Therefore, differential expression of sucrose synthase maybe key to the development of superior Pima fibers. Similarly, phenolic synthesis pathways (PAL and coumarin) are up-regulated during PCW and PCW/SCW overlapping stages in TM1, whereas only the PAL pathway is up-regulated from 21 to 24 dpa in developing Pima fibers. It was reported that coumarin inhibits cellulose synthesis in both PCW and SCW biogenesis in *in vitro *ovule cultures [13]. Based on this and our results, we therefore hypothesize that the up-regulation of coumarin biosynthesis pathway inhibits PCW extension in TM-1 relative to Pima fibers, in which coumarin biosynthesis is down-regulated. Increasing PAL activities result in elevated levels of ferulic acid (FA), which in turn, may lead to an increase in the level of cell wall-bound diferulic acid to curtail cell wall extensibility during the elongation stage [14]. In contrast, the up-regulation of PAL activities in the early stages of Pima SCW biogenesis, suggests a major role in the termination of the cell elongation phase, and in determining important properties such as fiber length. Similar results were obtained by Wakabayashi et al. [14] where they showed that abscisic acid curtails the extensibility of cell wall of wheat coleoptiles by decreasing cell wall-bound ferulic and diferulic acid.

## Conclusion

The novel application of double feature selection (DFS) combined with cluster analyses to mine independent microarray experiments proved effective in discovering new biologically relevant information not previously detected in microarray data. DFS revealed major biological processes that were linked to stage-specific events during cotton fiber development. Important metabolic processes, including sucrose metabolism and phenylpropanoid pathways, PAL and coumarin, are developmentally and differentially regulated in a genotype-specific manner as well. Based on these results, we propose that these processes in particular play a crucial role in a stage-specific manner that in turn, profoundly influence genotypic differences in fiber characteristics.

## Methods

Independent experiments of two representative genotypes of cultivated cotton species, *G. barbadense *L. cv. Pima S7 (Gb) and *G. hirsutum *L. cv. TM1 (Gh), were grown in a randomized block design under identical greenhouse conditions. Total RNA was extracted from single-celled developing fibers harvested at 5, 8, 10, 14, 17, 21 and 24 days post-anthesis (dpa). Developmental profiles of the cotton fiber transcriptome generated from an oligonucleotide microarray platform (NCBI-GEO Accession GPL6917) [15] using a double loop experimental design [16] included a dye swap hybridization strategy (Figure 1A). Direct microarray hybridizations (total 28) were performed per genotype to generate the transcriptome profiles. Custom Perl and Python scripts were employed to filter microarray raw data prior to normalization and statistical analysis using Linear Model for Microarray Data (LIMMA) in R (version 1.9.0) statistical software package [17]. Processed and raw data for Gb and Gh microarray experiments were deposited in NCBI-GEO in MIAME compliant format with the accession numbers GSE11689 and GSE11693, respectively.

### Data filtration and normalization

Data were filtered by applying the following steps: 1) Low quality spots were manually flagged, 2) Background noise on each sub-array was reduced by subtracting the background intensities mean from the spot signal intensities, 3) Corrected spot intensities lower than the mean of corrected buffer spots plus 3 standard deviations were excluded [18], 4) The absolute ratios (M = R/G) of dye swaps were inversed and the upper 30% quantile of divergent data points were removed. This cut-off value enhanced the correlation between dye swaps by increasing the linearity, and 5) Exclusion of all genes in which fewer than 60% of the data points did not pass all the filtration steps. In order to adjust for the effects that arise from variation inherent to microarray technology rather than biological differences in the RNA samples, filtered data was normalized using intensity-dependent normalization [19]. The robust scatter plot smoother 'Lowess' implemented in R package and LIMMA was used to perform a local A-dependent normalization. To make full use of within-array duplicated spots, LIMMA's pooled correlation method [20] was used to estimate the strength of the correlation between duplicated spots by fitting separate linear models to the expression data for each gene, but with a common value for between-replicate correlations.

### Data accuracy and reliability

The accuracy and reliability of microarray data was evaluated through the following steps: 1) The quality of microarray data, for every hybridization, was assessed before and after normalization via MA scatter plots of log_2_ratio versus log_2 _amplitude signals, 2) Dye bias was adjusted based on the correlation between Cy3 and Cy5 signals from self-hybridization control experiments, and 3) The reliability of the double loop design was determined by estimating the linearity between data derived from direct and indirect routes, and between two indirect routes in the double loop design (Figure 1).

### Linearization and significance analysis

Normalized high quality data fitted into seven linear models identified significant and differentially regulated genes at each developmental stage relative to each one of the other time points (global reference). The empirical eBayes method in LIMMA, which computes moderated t-statistics, moderated F-statistics, and log-odds of differential expression was applied to identify the significance of differential expression at each time point [21]. A correction for multiple testing using False Discovery Rates (FDR) [22] was used and significant changes in gene expression limited to p < 0.05. After identifying the significantly expressed genes in all linear models, the following data sets were created for each genotype: 1) Transcriptome profiles that include the expression coefficient relative to the expression at 5 dpa for all genes as calculated from all data points (8 per gene per time point) generated by the double loop hybridization design, and 2) Developmentally regulated profiles that include all significantly differentially expressed genes during fiber development relative to the expression at 5 dpa.

### Double feature selection

Feature selection has been used in many microarray data analyses in both supervised learning (classification) and unsupervised learning (clustering) contexts. Feature selection in our analysis is in the context of clustering. Double feature selection analysis was developed to discover the developmental time point(s) at which the greatest expressing distance (dissimilarity) occurred between the fiber developmental stages of the two genotypes, with simultaneous identification of the most differentially expressed genes between the transcriptomes of Pima and TM1 fibers. For this purpose, the expression matrix of the fiber transcriptome was used for each genotype. The novel double feature selection was developed as follows:

Let *gene(t, i) *denote a vector of the expression level of the *i*th gene for the genotype *t*, where *i *= 1, ..., 12063 and *t *= *Gh *or *Gb*. Define D(*i*) = || gene(*Gh*, *i*) – gene(*Gb*, *i*) || for *i *= 1, ..., 12,063, where || A ||^2 ^= sum of square of each entry in vector A. Sorting D values in a descending order gives a ranking for all of the genes in which top-ranked genes are more discriminating than lower ranked genes. A threshold was set at D ≥ 2. To determine the most discriminating time points (dpa) between the two fiber transcriptomes, all possible combinations of time points were investigated; that is, individual time points, as well as combinations of 2, 3, 4, 5, and 6 time points. The total number of combinations was as following:

$$\sum_{i=1}^{6}\left(\begin{array}{c} 6\\ i \end{array}\right)=63$$

For each combination, the summation of D(*i*), for *i *= 1, ..., 12,063 was computed. Time points in the combination that produces the highest summation of D's are the most discriminating time points. D was normalized by the number of time points in the combination. Two D's cannot be compared if their numbers of time points are different. That is, we cannot directly compare a D from a combination of two time points with a D from three time points. In our analysis, we considered the most discriminating combination of two time points. Additional theoretical work would be needed if one wants to pick the most discriminating time points from all possible time point combinations without relying on prior knowledge of cotton fiber development. To investigate the discriminatory power of the double feature selection analysis, the top discriminatory genes (D ≥ 2) between Pima and TM1 fiber transcriptomes were clustered using K-means, in which the Silhoutte score was used as a measure of the goodness [8], and combines both within-cluster cohesion and between-cluster separation. In detail, for the *i*^th ^object (gene), the silhouette score *s*_*i *_is defined as (*b*_*i*_*-a*_*i*_)/max(*a*_*i*_,*b*_*i*_) where *a*_*i *_is the average distance to all other objects in the cluster the *i*^th ^object belongs to, and *b*_i _is the minimum of average distances between the *i*^th ^object and all the objects any cluster not containing the *i*^th ^object. In the K-means clustering, each object is associated with a silhoutte score. The average of these scores ranges from -1 to 1 where 1 is a perfect clustering, 0 is a random clustering, and -1 is a bad clustering. Moreover, the expression distance matrix of the top discriminating genes was assessed for the presence of natural clusters using the singular value decomposition [23].

### Cluster analysis of developmentally regulated profiles

Differentially regulated genes in Pima and TM1 developing fibers were independently clustered using K-means. Similarly, the intersected genes between Pima developmentally regulated, TM1 developmentally regulated, and the top discriminating genes developed by double feature selection were clustered based on expression similarity. K-means clustering [24] using Pearson correlation as the similarity measure and 100 iterations was applied. This cluster analysis identified different stage-specific expression patterns in each gene set, and therefore linked the expression data with the various developmental stages in Pima and TM1 fibers.

### Biological relevance of the results

Genes were functionally analyzed in three steps: 1) The highest level of differential expression in each pattern was correlated to the corresponding fiber developmental stage and hence, to the major biochemical processes within the stage, 2) Major cellular activities, biological processes, and molecular functions were identified using GO annotation [25], and 3) Metabolic pathways and associated genes were identified, when possible, using KEGG pathways [26].

## Abbreviations

Days post-anthesis: dpa; distance: D; double feature selection: DFS; primary cell wall: PCW; secondary cell wall: SCW; singular value decomposition: SVD.

## Authors' contributions

MSA and EY conceived the study. MSA conducted Pima microarray experiments and data analysis. EY performed the double feature selection analysis. MSA performed cluster analysis. TAW provided TM1 microarray data and input into the concept. MSA drafted the manuscript with a contribution by EY, and revisions by TAW. All authors read and approved the final manuscript.

## Supplementary Material

Additional file 1 **Microarray expression data for Set 1 genes (125)**. The normalized expression values for Set 1 genes from Pima and TM1 developmental relative to the expression at 5 dpa. Genes are ordered according to the cluster number (as shown in Table 2). Functional descriptions were developed by BLASTing against NR unigene set (Genbank) and Arabidopsis (TAIR7) databases. Click here for file
